# The Identification and Quantitative Analysis of Unusual Keto-Carotenoids in Ripe Fruits of *Maclura tricuspidate* and Its Potential as a Valuable Source of Cryptocapsin

**DOI:** 10.3390/molecules27238317

**Published:** 2022-11-29

**Authors:** Jong-Kuk Kim, Dae-Woon Kim, Yoseph Asmelash Gebru, Han-Seok Choi, Young-Hoi Kim, Myung-Kon Kim

**Affiliations:** 1 Department of Food Science and Biotechnology, Jeonbuk National University, Jeonju 54896, Republic of Korea; 2 Institute of Jinan Red Ginseng, Jinan-gun 55442, Republic of Korea; 3 Department of Biological and Chemical Engineering, Mekelle University, Mekelle 231, Ethiopia; 4 Department of Agriculture and Fisheries Processing, Korea National College of Agriculture and Fisheries, Jeonju 54874, Republic of Korea

**Keywords:** *Maclura tricuspidata*, fruits, xanthophylls, cryptocapsin, capsanthin, ester forms, maturity stages

## Abstract

Ripe fruits of *Maclura tricuspidata* (MT) are used as food material and a natural colorant in Korea. Although MT fruits have a deep red color due to carotenoid-like pigments, their chemical nature has not been explored in detail so far. The present study aimed at elucidating the chemical structures and composition of carotenoids in MT fruits and changes at different maturity stages. Two carotenoids from saponified MT fruit extract were isolated using repeated silica gel column chromatography. Based on interpretations of spectroscopic data, these compounds were determined as keto-carotenoids, i.e., capsanthin (3,3′-dihydroxy-β,κ-caroten-6′-one) and cryptocapsin (3′-hydroxy-β,κ-caroten-6’-one), and the contents of individual carotenoids were quantified with HPLC based on calibration curves obtained from authentic standards. The contents of capsanthin and cryptocapsin in the sample of saponified MT fruits were 57.65 ± 1.97 µg/g and 171.66 ± 4.85 μg/g as dry weight base (dw). The majority of these keto-carotenoids in the MT fruits were present in esterified forms with lauric, myristic or palmitic acid rather than in their free forms. The results also showed that esterification of these compounds occurred starting from early stage (yellow-brownish stage) of maturation. Considering the high cryptocapsin content, MT fruits can be applied as a potentially valuable source of cryptocapsin for food and medicinal application as well as a source of provitamin A.

## 1. Introduction

It is well known that pigment-rich foods such as colorful fruits, berries and vegetables play important roles in the prevention of chronic diseases such as cancer, obesity, diabetes and hypertensive, cardiovascular and neurodegenerative disorders as well as nutritional supplements [1,2]. Among these pigments, carotenoids are responsible for yellow, orange and red colors in a wide variety of living organisms such as plants, algae, microorganisms and some animal tissues. Some carotenoids have been found to have important nutritional, physiological and pharmacological functions. More recently, carotenoids are receiving considerable attention due to their various health benefits including antioxidant, anti-cardiovascular disorder and anticarcinogenic potentials and the prevention of the risk of age-related macular degeneration and activation of the immune system [3,4,5,6]. Carotenoids can be classified into two major groups as follows. Carotenes are comprised of only carbon and hydrogen while oxygenated carotenoids (commonly known as xanthophylls) contain oxygen with carbon and hydrogen in their molecular structures. Lutein, zeaxanthin, β-cryptoxanthin, violaxanthin, astaxanthin and capsanthin are representative compounds belonging to xanthophylls found in nature. Xanthophylls exist in fruits and vegetables both in the free or esterified forms (acylated with fatty acids) [7,8]. In fruits and vegetables, the esterification degree of xanthophyll is species-specific [7]. Xanthophyll esters are abundant in fruits such as papaya, mango, orange and in petals of some flowers whereas no xanthophyll esters are detected in green vegetables such as spinach and broccoli [7,8]. Particularly, capsanthin, capsorubin and cryptocapsin are xanthophylls containing 6-oxo-κ end-group (keto-carotenoids) in their molecules. These constituents were mainly found among *Capsicum* spp. including paprika and red pepper, and rarely Central American fruits such as *Pouteria sapota*, *Cionosicyos macranthus* and *Carludovica palmata* [9,10,11,12]. They were also suggested to have potential health benefits due to their antioxidant, cardiovascular and anti-amyloid aggregation activities [13,14].

On the other hand, *Maclura tricuspidata* (Carr.) Bur. which belongs to the Moraceae family is a deciduous tree distributed in East Asian region including China, Japan and Korea. The different parts including leaves, root, stem and fruits of this plant have been used in traditional folk medicine to treat jaundice, hepatitis, neuritis and inflammation [15]. In particular, the ripe MT fruits which have a distinct red color has been traditionally used for the preparation of concentrated juice, vinegar, jam and alcoholic beverages as well as food colorants in Korea. Although fully ripe MT fruits have a deep red color, which is believed to be due to carotenoid-like pigments, their chemical natures and compositions are not fully elucidated until now. A previous study reported that the carotenoids of MT fruits were mainly composed of β-carotene, neo-β-carotene, polycopene and ruboxanthin with α-carotene, lycopene, zeaxanthin and lutein as trace compounds based on only TLC R*f* values and UV-Vis. absorption profiles [16]. However, the information on a definite chemical structures, and their compositions of carotenoid pigments in MT fruits are still poor. Furthermore, it is well known that oxygenated carotenoids in fruits and vegetables are present in free and esterified forms or in either of the two forms. But little is known about how these pigments are present in any forms in the MT fruits.

Therefore, the objective of this study was to explore the chemical structures of main carotenoids in MT fruits and investigate changes in compositions of free and esterified carotenoids at different maturity stages.

## 2. Results and Discussion

### 2.1. Analysis of Free and Esterified Carotenoids in MT Fruits

**Figure 1:**
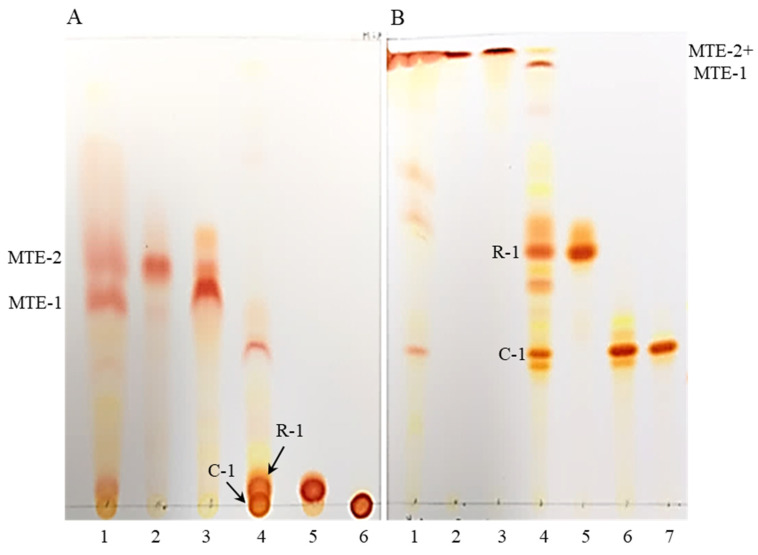
TLC profiles of total extract, MTE-1 and MTE-2 fractions isolated from solvent extract by open column chromatography. 1, un-saponified extract; 2, un-saponified MTE-2 fraction; 3, un-saponified MTE-1 fraction; 4, saponified extract; 5, saponified MTE-2 fraction; 6, saponified MTE-1 fraction; 7, capsanthin (authentic standard). TLC solvents; (**A**), *n*-hexane: acetone (95:5, *v*/*v*); (**B**), *n*-hexane: acetone (70:30, *v*/*v*).

In this study, analyses by thin layer chromatography (TLC) and high performance liquid chromatography (HPLC) were performed to investigate whether carotenoids in the MT fruits are present in free forms (carotenes or free xanthophyll) or in esterified forms (esterified xanthophyll). As shown in Figure 1A, two large fractions (MTE-1 and MTE-2), that have different R*f* values were detected from an un-saponified extract in the TLC analysis using *n*-hexane: acetone (95:5, *v*/*v*) as a developing solvent. Both fractions were isolated by preparative thin layer chromatography (PTLC) and silica gel column chromatography. Thereafter, when three samples (total extract, MTE-1 and MTE-2 fractions) were subjected to saponification with alkaline solution (10% KOH in MeOH), two new spots, that were not detected in un-saponified samples were appeared (Figure 1B). These results indicate that esterified xanthophylls were hydrolyzed into free forms under alkaline condition. These results also suggest that most of carotenoids in the ripe MT fruits are xanthophyll carotenoids and those are present in esterified forms rather than in free forms. Furthermore, when MTE-1 and MTE-2 fractions were analyzed by TLC using a more polar solvent system (*n*-hexane: acetone = 70:30), MTE-1 and MTE-2 were detected as a single spot (C-1 and R-1) with different R*f* values, respectively. These results also demonstrate that chemical structures of C-1 and R-1 in saponified products are different from each other.

**Figure 2:**
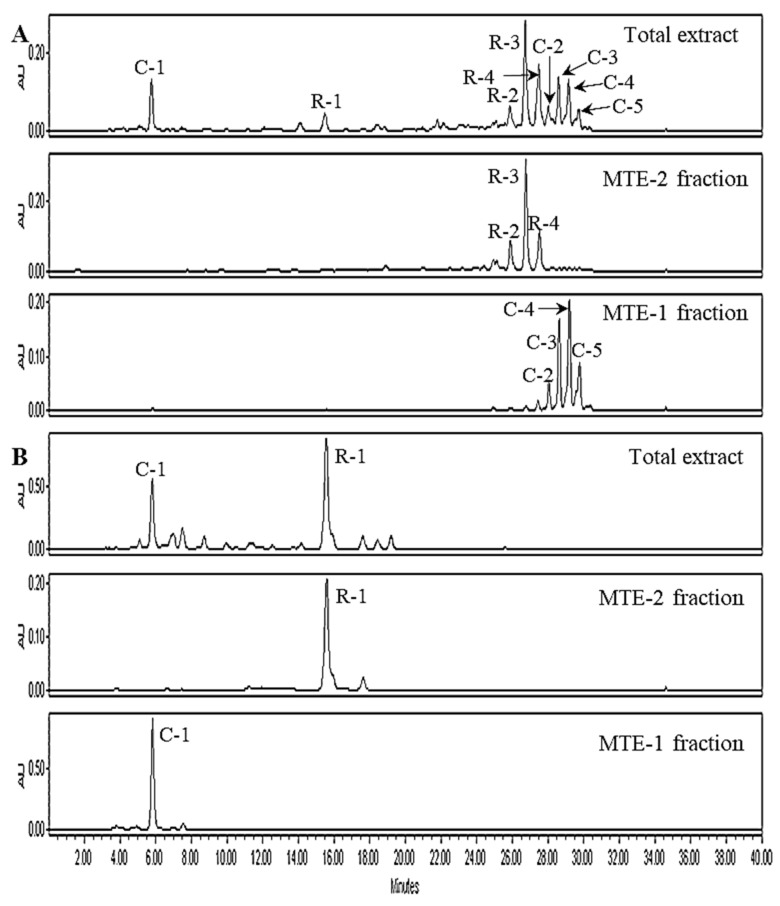
HPLC profiles of total extract, MTE-1 and MTE-2 fractions isolated from ripe MT fruits. (**A**), unsaponified; (**B**), saponified. HPLC conditions: column, Sunfire C_18_ (250 × 4.6 mm, 5 μm, Agilent Technologies); mobile phase, acetone (**A**) and deionized water (**B**); gradient condition, A:B = 80:20 (0–15 min), 95:5 (15–20 min), 100:0 (20–30 min) and 80:20 (30–40 min); flow rate, 1.0 mL/min; detection, PDA (450 nm).

The HPLC chromatograms of un-saponified total extract, MTE-1 and MTE-2 fractions are shown in Figure 2A. A total of 9 different peaks were detected in total extract, while 4 peaks (peak no C-2–C-4) and 3 peaks (peak R-2–R-4) in MTE-1 and MTE-2 fraction were detected, respectively. However, MTE-1 and MTE-2 fractions showed different HPLC profiles, which are expected to be due to differences of molecular backbone, esterification degree (mono- or di-ester forms,) or chain length of acylated fatty acids.

Saponification of esterified xanthophyll generates the parent free xanthophylls and free fatty acids. The saponified samples of MTE-1 and MTE-2 fractions were detected as a single peak (C-1 and R-1) with different retention time in HPLC analyses, respectively, while it was detected as a mixture of two compounds in total extract. These results also suggest that the xanthophylls in MT fruits are presented as ester mixtures of C-1 and R-1. Accordingly, it is necessary to identify the chemical structures of the two xanthophylls (C-1 and R-1) detected in the saponified MT fruits.

### 2.2. Structure Elucidation of C-1 and R-1 from Saponified Extract

**Figure 3:**
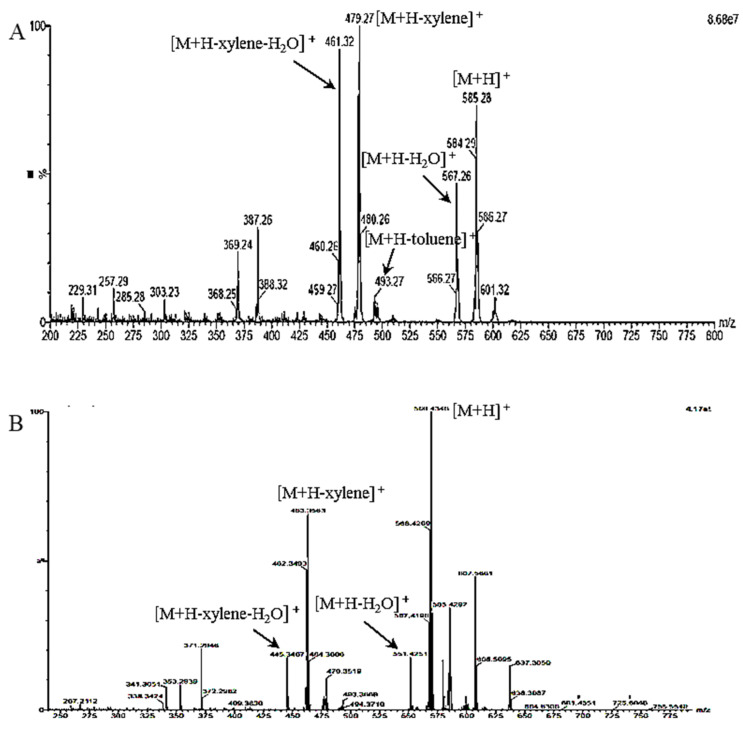
Mass spectra of C-1 (**A**) and R-1 (**B**) isolated from saponified extract of ripe MT fruits. (**A**) positive APCI-MS spectrum; (**B**) positive APCI-Q-TOF-MS/MS spectrum.

**Figure 4:**
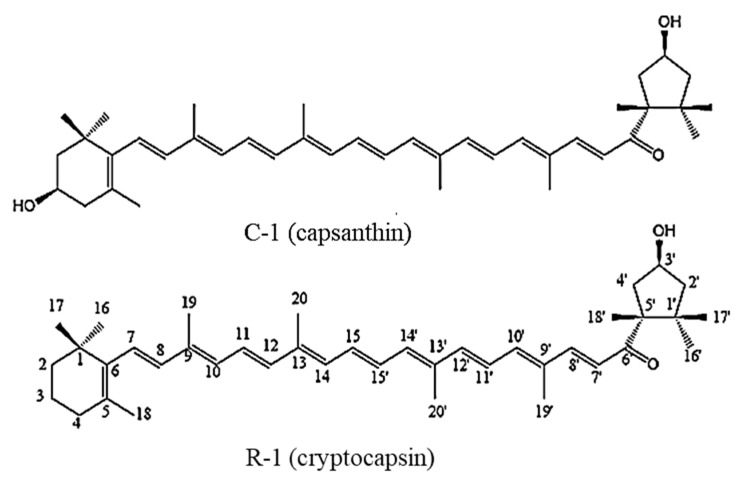
Chemical structures of major xanthophyll isolated from saponified extract of MT fruits.

Two compounds (C-1 and R-1) were isolated from saponified MT fruit extract by silica gel column chromatography and flash chromatography using reversed-phase C_18_ cartridge. Compound C-1 was isolated as a deep red amorphous solid. The ultraviolet/visible (UV-Vis.) spectrum of C-1 showed maximum absorption wavelength (λ_max_) at 468.5 nm (in acetone). The spectrum by atmospheric pressure chemical ionization–mass spectrometer (APCI-MS) in the positive mode of C-1 as shown Figure 3A was characterized by the presence of a protonated molecule [M+H]^+^ at *m*/*z* 585.28, which was constituent with molecular formula C_40_H_56_O_3_ (MW = 584.88). In addition, fragment ions of *m*/*z* 567.26 [M+H−H_2_O]^+^, 493.27 [M+H−toluene]^+^, 479.27 [M+H−xylene]^+^ and 461.32 [M+H−xylene−H_2_0]^+^, 387.26 [M+H−toluene−xylene]^+^ and 369.24 [M+H−toluene−xylene−H_2_0]^+^ were observed. These ions in the APCI spectrum can be regarded as fragment ions formed by eliminations of water (−*m*/*z* 18), toluene (−*m*/*z* 92) or xylene (−*m*/*z* 106) from the polyene chain in molecule [17,18,19,20]. The ^1^H-NMR spectrum of C-1 showed 10 methyl signals at δ 0.84 (H-17′), 1.07 (H-16), 1.21 (H-16′), 1.26 (C-17). 1.36 (H-18′), 1.74 (H-18), 1.96 (C-20), 1.97 (H-19′, C-20′) and 1.99 (H-19) (all 3H, s) with several proton signals derived from the polyene chain as shown in Table 1. In the ^13^C-NMR spectrum of C-1, a total of 40 carbon signals were observed. Therefore, it can be predicted that C-1 is a carotenoid constituted of 40 carbons. The ^13^C-NMR spectrum of C-1 showed 10 methyl signals at δ 12.79 (C-19), 12.84 (C-19′), 12.93 (C-20′), 12.96 (C-20), 21.32 (C-18′), 21.63 (C-18), 25.08 (C-16′), 25.88 (C-17′), 28.74 (C-16) and 30.27 (C-17). Additionally, the signals by the three oxygen functions were observed in the ^1^H- and ^13^C-NMR spectra, Among them, one was ascribed to a carbonyl group (δ_C_ 203.05) attached to C-6′ position of polyene chain; the other two are due to hydroxyl groups attached in positions of C-3 and C′-3 at δ_H_ 3.99 (1H, m), δ_C_ 65.13 in the β-ring and δ_H_ 4.52 (1H, m), δ_C_ 70.38 in the κ-ring. All the spectroscopic data of C-1 were in agreement with data of capsanthin reported in the literature [18,21,22]. From these results, C-1 was determined as capsanthin (3,3′-dihydroxy-β,κ-carotene-6′-one) as shown in Figure 4. Although capsanthin in free and esterified forms is known as a major pigment in the fruits of *Capsicum* spp. such as red pepper and paprika [9,23,24,25], the presence of esterified capsanthins in MT fruits was revealed for the first time in this study.

Compound R-1 showed λ_max_ at 468.5 nm (in acetone), which was similar to that of C-1. These results indicate that R-1 and C-1 have a similar chromophore. The UV-Vis. absorption spectra of carotenoids having keto-κ end-ring in its molecule can be characterized by a single absorption maximum due to the conjugation of the carbonyl group with the polyene chain [10,12,22]. Mass spectrum of R-1 in positive mode by quadrupole time-of-flight mass spectrometry (Q-TOF-MS) was characterized by the presence of a protonated molecule [M+H]^+^ at *m*/*z* 569.4346, which was constituent with molecular formula C_40_H_56_O_2_ (MW 568.87). The molecular weight of R-1 was 16 amu less than that of C-1, which is presumed to be due to the elimination of one hydroxyl group from capsanthin (C-1). Additionally, fragment ions of *m*/*z* 551.4251 [M+H−H_2_O]^+^, 463.3563 [M+H−xylene]^+^ and 445.3467 [M+H−xylene−H_2_O]^+^ were observed (Figure 3B). These fragment ions in the MS spectrum can be regarded as ions formed by eliminations of water (−*m*/*z* 18), toluene (−*m*/*z* 92) or xylene (−*m*/*z* 106) in the polyene chain of the carotenoid [10,12,14,18,19].

Either way, the molecular weight of R-1 was confirmed to be identical with those of cryptocapsin and 3′-deoxycapsanthin belonging to keto-carotenoids [10,14,22]. In the ^1^H-NMR spectrum of R-1, 14 olefin methine signals were observed at ó 6.73–6.12 and 10 singlet methyl signals at ó 0.84, 1.03, 1.21, 1.25, 1.37, 1.72, 1.96, 1.98 (×2) and 1.99. In addition, two oxygen functions were observed in the ^1^H- and ^13^C-NMR spectra. One of them was due to a carbonyl group (δ_C_ 202.95) attached to the C-6′ position of the polyene chain, and the other was due to the hydroxyl group (δ_H_ 4.52, δ_C_ 70.36) at C-3′ on the κ-ring. In the ^13^C-NMR spectrum, the 10 signals due to methyl groups were observed at δ 12.79 (C-19), 12,84 (C-19′), 12.93 (C-20′), 12.96 (C-20), 21.32 (C-18′), 21.63 (C-18), 25.08 (C-16′), 25.88 (C-17′), 28.74 (C-16) and 30.27 (C-17. The ^1^H-NMR and ^13^C-NMR data of R-1 are the same as those of C-1, except for the signals of the β-end ring (C-1–C-5). The signals at the β-end ring were observed at ó_C_ 34.28 for C-1, ó_C_ 39.68, ó_H_ 1.47 (2H, m) for C-2, 19.26, 1.64 (2H, m) for C-3, 33.12, 2.02 (2H, m) for C-4 and ó_C_ 129.50 for C-5, while those of β-end ring in C-1 were observed at ó_C_ 37.4 for C-1, ó_C_ 48.42, ó_H_ 1.48 (2H, m) for C-2, 65.13, 3.99 (2H, m) for C-3, 42.54, 2.39 (1H, dd, *J* = 17.2, 4.8 Hz) for C-4 and ó_C_ 126.26 for C-5. R-1 was assigned based on DEPT, ^1^H-^1^H COSY, HMBC and HSQC measurements and by comparison of NMR spectral data with cryptocapsin and 3′-deoxycapsanthin reported in the literature [10,14]. Correlations between neighboring proton signals were observed in the COSY spectrum, and the J_1_ correlations between carbon signals and proton signals were confirmed in the HSQC spectrum. The J_2_ and J_3_ correlations between carbon signals and proton signals were also confirmed in the HMBC spectrum. Consequently, the chemical shift and coupling pattern of the R-1 were found to be in agreement with previous literature that reported cryptocapsin [10,14]. Thus, R-1 was elucidated as cryptocapsin (3′-hydroxy-β,κ-caroten-6′-one) (Figure 4).

The majority of naturally occurring carotenoids have unsaturated substituted six-membered rings (β-end group), whereas carotenoids containing five-membered rings (κ-end group) are relatively rare. Capsanthin and capsorubin are the representative carotenoids containing a κ-ring as an end group, and these compounds are mainly distributed in *Capsicum* spp. fruits such as red pepper and paprika [26]. Cryptocapsin was identified from paprika [9,27,28] and red mamey [10,11,12]. To the best of our knowledge, only one paper on carotenoids of MT fruits has been reported until now [16]. The result reported the presence of phytofluin, α- and β-carotene, neo-β-carotene, lycopene, polycopene, zeaxanthin, ruboxanthin and lutein as carotenoids of MT fruits based on the absorption maxima, R*f* and visual color of the isolated compounds by preparative TLC (PTLC). However, contrary to previously reported results, the esterified compounds of capsanthin and cryptocapsin are the main compounds responsible for the red color of ripe MT fruits.

### 2.3. The Identification of Esterified Carotenoids from Un-Saponified MTE-1 and MTE-2 Fractions

**Figure 5:**
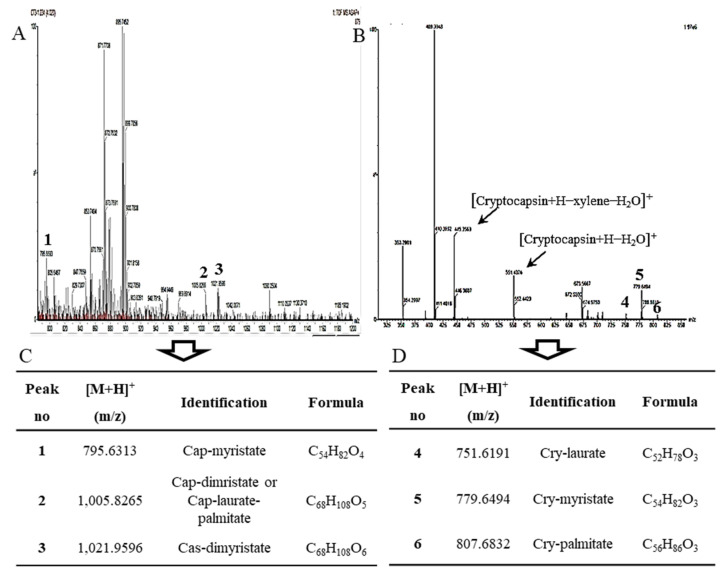
Positive APCI-QTOF-MS analysis and identification of MTE-1 (**A**) and MTE-2 (**B**) fractions isolated from un-saponified extract of MT fruits. Cap, capsanthin; Cas, capsorubin; Cry, cryptocapsin. Tables below were keto-carotenoid esters identified from un-saponified MTE-1 (**C**) and MTE-2 fractions (**D**).

To explore the composition of xanthophyll esters in the MTE-1 and MTE-2 fractions, they were analyzed by APCI-Q-TOF-MS without saponification as shown in Figure 5. The molecular formulas of peak nos. 1, 2 and 3 detected in MTE-1 fraction (Figure 5A) were determined to be C_54_H_82_O_4_, C_68_H_108_O_5_ and C_68_H_108_O_6_, respectively, from the molecular ion peaks [M+H]⁺ at *m*/*z* 795.6313 (calc. for C_54_H_83_O_4_, 795.6291, error value ∆ = +2.8 ppm), 1005.8266 (calc. for C_68_H_109_O_5_, 1005.275, ∆ = −0.9 ppm) and 1021.8196 (calc. for C_68_H_109_O_6_, 1021.8224, ∆ = 2.7 ppm) in the positive APCI-QTOF-MS. These mass spectral data were consistent with those of capsanthin (Cap)-myristate, Cap-dimyristate or Cap-laurate-palmitate and capsorubin-dimyristate [24]. In addition, the molecular formulas of peak nos. 4, 5 and 6 in MTE-2 fraction (Figure 5B) were determined to be C_52_H_78_O_3_, C_54_H_82_O_3_ and C_56_H_86_O_3_, respectively, from the molecular ion peaks [M+H]⁺ at *m*/*z* 751.6191 (calc. for C_52_H_79_O_3_, 751.6182, ∆ = +1.2 ppm), 779.6514 (calc. for C_54_H_83_O_3_, 779.6495, ∆ = +2.4 ppm) and 807.6832 (calc. for C_56_H_87_O_3_, 807.6808, ∆ = +3.0 ppm) in the positive mode. These mass spectral data were also consistent with those of cryptocapsin (Cry)-laurate, Cry-myristate and Cry-palmitate [29]. The presence of these keto-carotenoid esters in MT fruits were revealed for the first time in this study, although the esters of capsanthin and crytocapsin identified in this study were previously reported in red pepper pod (*Capsicum annum* L.) [7,24] and the tropical fruits, mamey sapote and mamey rojo (*Pouteria sapota*) [11,29]. In fruits and vegetables, the esterification degree and composition of xanthophylls are species-specific [7]. Green vegetables such as spinach, broccoli, parsley, sprouts and unripe fruits that have chloroplasts as the plastids do not contain xanthophyll esters [8,30]. In contrast, xanthophyll esters are abundant in ripe fruits such as red pepper; paprika; sea buckthorn; orange; mandarin; apricot; apple and Central American fruits such as mamey sapote (*P. sapota*), *Cionosicyos macranthus*, *Carludovica palmate* and marigold flower [8,13,29,31,32,33,34,35,36]. However, most of the capsanthin and cryptocapsin in MT fruits was found to exist in esterified forms with fatty acids.

### 2.4. Comparison of Keto-Carotenoid Profiles at Different Maturity Stages

**Figure 6:**
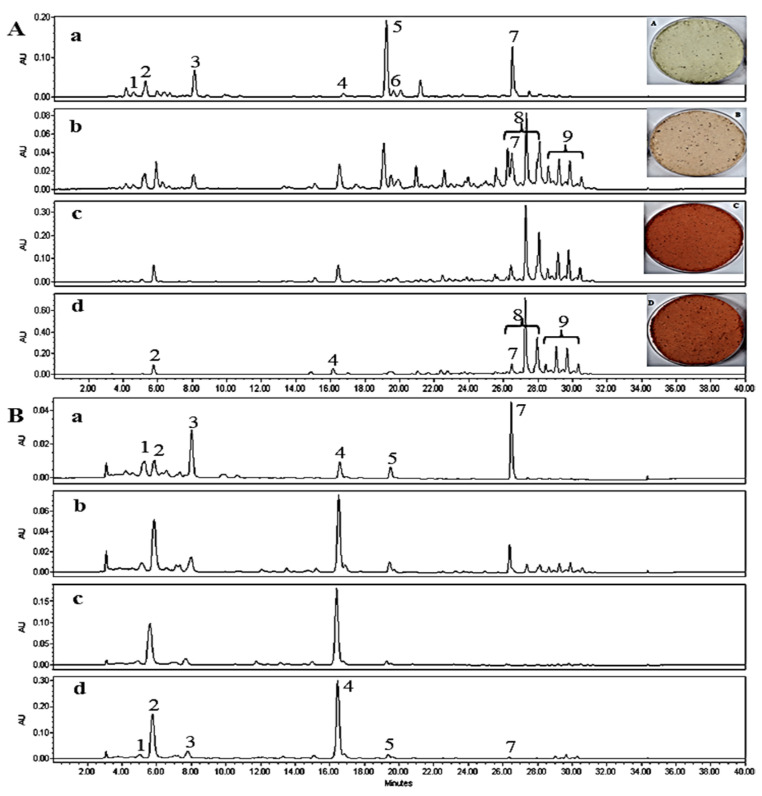
Comparison of HPLC profiles of un-saponified (**A**) and saponified (**B**) extracts of MT fruits at different maturity stages: a, immature (pale green); b, premature (brownish); c, fully mature (red); d, overmature (deep red); 1, capsorubin; 2, capsanthin, 3, lutein; 4, cryptocapsin; 5, unidentified; 6, β-cryptoxanthin; 7, β-carotene; 8, cryptocapsin esters; 9. capsanthin esters.

The external color of MT fruits changes from green to deep red during fruit development and ripening, which is due to changes in the concentration and composition of chlorophylls and carotenoids. The total carotenoid content (33.75 ± 1.53 mg β-carotene equivalent (CAE)/g) of MT fruits at the fully mature stage was about 4.5 times higher than that at the immature stage (7.46 ± 0.44 mg β-CAE/g) [37]. The maturation stages of MT fruits also affect the composition of carotenoids and esterification degree of xanthophylls. The noticeable increase in total carotenoid content and esterified carotenoids with fruit maturation has been observed in various types of fruits and berries such as red pepper, paprika, sweet oranges and goldenberry [23,28,31,38,39,40]. Figure 6 shows HPLC profiles before and after the saponification of MT fruit extract as an influence of maturity stage. β-Carotene (peak 7), unidentified compound (peak 5) and lutein (peak 3) were detected as main carotenoids in immature stage (pale green).

As maturation progresses, these compounds gradually decreased, while esterified carotenoids (peaks 8 and 9) markedly increased (Figure 6A). Additionally, the results showed that capsantin and crypotocapsin esters in the MT fruits accumulated as acylated forms with lauric, myristic and palmitic acid. Table 2 shows the changes in compositions and contents of carotenoids of MT fruits at different maturity stages. These results indicate that xanthophylls start to increase in the premature stage, and thereafter, their contents increase continuously with progressive maturation, but no significant change was observed in the composition of the esterified xanthophylls.

In contrary, lutein and β-carotene decrease continuously as maturation progressed. A similar trend was also observed in the maturation stage of red pepper, paprika, orange and other fruits [28,31]. It has been previously reported that cryptocapsin is biosynthesized from β-carotene via β-cryptoxanthin as an intermediate metabolite during maturation in fruits [10,41]. The rapid decrease in β-carotene during the progression of maturation observed in this study might be associated with a sharp increase in cryptocapsin. Particularly, the contents of capsanthin (57.65 ± 1.97 µg/g) and cryptocapsin (171.66 ± 4.85 µg/g) in the fully mature stage after saponication showed about 30.5 and 50.2 increases compared with those of the immature stage (1.37 ± 0.14 µg/g and 4.07 ± 0.24 µg/g), respectively. Additionally, the contents of capsanthin and cryptocapsin in the fully mature and overmature stages were significantly higher in the saponified extracts than in the un-saponified extracts. These increases are due to the hydrolysis of ester forms of capsanthin and cryptocapsin into free forms under the alkaline condition. Although the cryptocapsin content in some red pepper (*C. annum* var. Km 622) has been reported to be about 770 µg/g, its usual general concentration in most red pepper and paprika is in trace amounts (<10 µg/g) [9,28]. These results letdus to conclude that ripe MT fruits can be used as a valuable source of cryptocapsin due to its high content.

## 3. Materials and Methods

### 3.1. Plant Materials

A total of 10 kg of fresh *Maclura tricuspidata* (MT) fruits were purchased from a local farm located in Milyang city (Gyeongsangnam-do, Republic of Korea) in late October 2020 and authenticated by Professor Byung-Kil Choo (Department of Crop Agriculture and Life Science, Jeonbuk National University, Republic of Korea). Voucher specimen (FL-202001) was deposited in the Laboratory of Fermentation Technology (Prof. Myung-Kon Kim, Jeonbuk National University, Republic of Korea). The fruits were sorted into four different groups based on their exterior color, i.e., immature (pale green), premature (yellow-brownish), mature (red) and ovemature (deep red) stages [37]. The sorted samples were then washed with distilled water, followed by freeze-dryng for 7 days. Samples were powdered using a household grinder (Hanil SFM-700SS, Yeongdeungpo-gu, Seoul, Republic of Korea). The powdered samples were kept in airtight plastic bags and stored in a freezer (−20 °C) until use.

### 3.2. Reagents

Butylated hydroxytoluene (BHT), β-carotene and lutein were purchased from Sigma-Aldrich Corp. (St. Louis, MO, USA). Authentic capsorubin, capsanthin and β-cryptoxanthin were purchased from Extrasynthese (Impasse Jacquard, Genay Cedex, France). Deuterated chloroform (CDCl_3_), tetramethylsilane (TMS), silica gel F_254_ plate for TLC (0.25 mm thickness), PTLC (1 mm thickness) and silica gel (230–240 mesh) for open column chromatography were purchased from Merck KGaA (Darmstadt, Germany). Acetonitrile, acetone and tetrahydrofuran (HPLC grade) were purchased from Advantor Performance Materials Korea (Suwon, Gyeonggi-do, Republic of Korea). Deionized water was prepared using a water purification system (model New Human Power I, Human Corp., Songpa-ku, Seoul, Republic of Korea). The other reagents used were purchased from commercial sources (Daihan Scientific Co., Ltd., Gangwon-do, Republic of Korea).

### 3.3. Extraction of Carotenoids

200 g of powdered MT fruits was extracted with 800 mL of a mixture of *n*-hexane and acetone (70:30, *v*/*v*) using an ultrasonicator (Hwa Shin Instrument Co., Seoul, Republic of Korea) at room temperature for 20 min and centrifuged at 5000× *g* for 20 min. The residue was extracted twice more with same solvent mixture followed by centrifugation as described above. The supernatant was combined and vacuum evaporated at 40 °C to obtain the extract (16.6 g, yield; 8.3%). For saponification, 10 g of the extract containing carotenoids was dissolved in acetone (30 mL) and 10% methanolic KOH solution (50 mL). The mixture was saponified for 2 h at 65 °C with gentle shaking [42,43]. The termination of saponification reaction was monitored with TLC (*n*-hexane: acetone = 95:5, *v*/*v*) until carotenoid esters disappeared. The saponified extract was transferred to separatory funnel together with saturated sodium chloride (50 mL) and was subsequently extracted with diethyl ether (150 mL × 3). The ether layer was washed three times with distilled water (each 50 mL) to remove excess alkali and residual acetone. The ether extract was dried over anhydrous sodium sulfate for 12 h and filtered. The filtrate was subjected to dryness in a vacuum evaporator to obtain the crude saponified extract.

### 3.4. Isolation of Esterified Carotenoid Fractions from Un-Saponified Extract

The un-saponified extract was dissolved in a small volume of acetone and was applied as a line on a silica gel PTLC plate (20 cm × 20 cm, thickness 1.0 mm) and was developed with 5% acetone in *n*-hexane. Two major bands with distinct red color were obtained on repeated chromatography on PTLC plates with same solvent system. The bands were scraped off into a beaker containing acetone as the eluent. The acetone extracts were subjected to dryness in a vacuum pressure to obtain two esterified carotenoid fractions (named MTE-1 and MTE-2). The two fractions were further purified by silica gel column chromatography with stepwise gradient of *n*-hexane–ethyl acetate, respectively.

### 3.5. Isolation of Capsanthin and Cryptocapsin from Saponified Extract

The saponified MT fruit extract (10 g) was separated by silica gel column chromatography (20 × 5 cm) and eluted with stepwise gradient (100:0 to 50:50) of *n*-hexane–acetone to yield eight fractions (MF1-1–MF1-8). Fraction MF1-6 was eluted with *n*-hexane–acetone (75:25) and was further subjected to silica gel chromatography with a stepwise gradient of *n*-hexane–ethyl acetate (80:20 to 30:70) to afford 50 sub-fractions (MF2-1–MF2-50). MF2-17–22 (1.79 g) and MF2-37–41 were re-chromatographed on silica gel with a stepwise gradient of *n*-hexane–ethyl acetate (80:20 to 30:70) to obtain R-1 and C-1, respectively. Further purification of C-1 and R-1 were performed with a Reveleris flash chromatography system (Buchi Corp., Lukens Drive, New Castle, DE, USA) using a Reveleris^®^ C_18_ cartridge (40 g) with UV (450 nm) and evaporative light scattering detector (ELSD) with a gradient elution (0 → 100%) of water and acetone. The eluates were evaporated under reduced pressure to obtain purified C-1 (0.39 g) and R-1 (0.55 g). The two compounds were obtained as amorphous powder with deep red color.

C-1: UV-Vis. (in acetone) λ_max_ 468.5 nm; positive APCI-MS (*m*/*z*); 585.28 [M+H]^+^ (C_40_H_57_O_3_) (calc. for 585.8708), 567.26 [M+H–H_2_O]^+^, 493.27 [M+H−toluene]^+^,479.27 [M+H−xylene]^+^, 461.32 [M+H−xylene−H_2_O]^+^, 387.26 [M+H−toluene−xylene]^+^, 369.24 [M+H−toluene−xylene–H_2_O]^+^; ^1^H- (500 MHz, CDCl_3_) and ^13^C-NMR (125 MHz, CDCl_3_) see Table 1.

R-1: UV-Vis. (acetone) λ_max_ 468.5 nm; Positive ESI-MS *m*/*z* 569.3 [M+H] ^+^; high resolution positive APCI-Q-TOF-MS *m*/*z* 607.5661 [M + K]^+^, 585.4927 [M + OH]^+^, 569.4346 [M+H]^+^ (calc. for C_40_H_57_O_2_ 569.4354, error value, ∆ = +1.4 ppm), 551.4251 [M+H−H_2_O]^+^, 463.3563 [M+H−xylene]^+^, 445.3467 [M+H−xylene−H_2_O]^+^, 371.2946 [M+H−xylene−toluene]^+^; ^1^H-(500 MHz, CDCl_3_) and ^13^C-NMR (125 MHz, CDCl_3_) see Table 1.

### 3.6. TLC, HPLC and Q-TOF-MS Analysis

TLC was performed on silica gel 60 F_254_ with *n*-hexane: acetone (95:5, *v*/*v*) or *n*-hexane: acetone (70:30) as the developing solvent. The spots on the TLC were detected visually or by heating at 110 °C for 10 min after spraying 10% sulfuric acid in ethanol. HPLC analysis was performed using a HPLC system (Waters, Milford, MA, USA) equipped with a 600E system controller, a 717 plus autosampler, and a 996-photodiode array detector (450 nm) with a Sunfire C_18_ column (250 × 4.6 mm, 5 μm; Agilent Technologies, Inc., Santa Clara, CA, USA). The mobile phase consisted of acetone (A) and deionized water (B). The ratio of mobile phase was maintained at A:B = 80:20 (0–15 min), 95:5 (15–20 min), 100:0 (20–30 min) and 80:20 (30–40 min) at a flow rate of 1.0 mL/min. Each carotenoid was identified by comparing HPLC retention time and UV profile with those of authentic standards. MS analysis was performed on Agilent 1100 LC-MS system (Agilent Technologies, Santa Clara, CA, USA) or a Q-TOF-MS (Synapt G2-Si HMDS, Waters Corp., Milford, MA, USA) with APCI in positive mode. The conditions for MS analyses were: drying gas N_2_, flow rate 12 L/min, cone gas temperature 350 °C, nebulizer pressure 50 psi and capillary voltage 4.0 kV.

### 3.7. Preparation of Standard Solution

Each 1.00 mg of authentic standards (capsorubin, lutein, β-cryptoxanthin and β-carotene) and the isolated compounds (capsanthin and cryptocapsin) from MT fruits were dissolved in 2 mL of mixture of acetone: tetrahydrofuran (80:20, *v*/*v*) as a stock solution. Working standard solutions were serially diluted with same solvent to obtain a concentration of 0.781–100 μg/mL (8 points). The working solutions were prepared before HPLC analysis. Calibration curve of carotenoid was obtained by plotting the peak area of individual carotenoid against the concentration, and the content was calculated as micrograms per gram (μg/g, dw).

### 3.8. Carotenoid Contents at Different Maturity Stages

The powdered samples for MT fruits (2.0 g) were extracted with a mixture of *n*-hexane and acetone (20 mL at a ratio of 70:30, *v*/*v*) containing 0.1% BHT [20,44] as an antioxidant with an ultrasonicator (Hwa Shin Instrument Co., Seoul, Republic of Korea) at room temperature for 20 min and centrifuged at 4500 rpm for 20 min. The residues were extracted twice more with 20 mL of the same solvent mixture followed by centrifugation as described above. The supernatants were combined and evaporated under reduced pressure. The residue was dissolved in 2 mL of acetone: tetrahydrofuran (80:20, *v*/*v*) and diluted when necessary. HPLC analysis was performed according to the methods described above.

### 3.9. Spectroscopic Analysis

Maximum absorption wavelength (λ_max_) of isolated compounds was obtained in acetone with a UV-Vis. spectrophotometer (UV-188, Shimadzu Corp., Kyoto, Japan). The NMR measurements were carried out using a JEOL JNM-ECXZ500R spectrometer (Tokyo, Japan) operating at 500 MHz and 125 MHz for ^1^H- and ^13^C-NMR using CDCl_3_ as a solvent and TMS as an internal standard.

### 3.10. Statistical Analysis

All experiments were performed in independent triplicate experiments unless otherwise indicated, and the results are presented as a mean ± standard deviation (SD). The statistical analysis was conducted with SPSS (ver. 10.1) for Windows and a one-way analysis of variance (ANOVA). Duncan’s multiple range tests were carried out to test any significant differences. Values with *p* < 0.05 were considered as significantly different.

## 4. Conclusions

Recently, carotenoids have received much attention due to their health benefits, and the global demand for bioactive carotenoids is expected to increase steadily in the future. Therefore, research on the exploration of new bioactive carotenoids or natural sources rich in bioactive carotenoids is of paramount importance globally. In the present study, it was revealed for the first time that most of the carotenoids in MT fruits are composed of ester mixtures of capsanthin and cryptocapsin. Capsanthin has been reported to have therapeutic potentials including antioxidant, chemopreventive, antitumor, skin photo-protective, anti-inflammatory and antidiabetic properties. On the contrary, little is known about the health benefits of cryptocapsin except for antioxidant, and provitamin A activity and anti-amyloidogenic potential. Accordingly, further exploration is necessary to promote the use of cryptocapsin in the food and pharmaceutical industries. Although capsanthin and cryptocapsin are commonly found among *Capsicum* spp. fruits including red pepper and paprika, their contents in these fruits are present in trace amounts (<10 µg/g). Considering the high cryptocapsin content, we suggest that ripe MT fruits have potential as an excellent source of cryptocapsin for food and pharmaceutical application as well as a source of provitamin A.

## Figures and Tables

**Table 1:** NMR spectral data for compounds C-1 and R-1 ^a^.

Carbon No	C-1 (Capsanthin)		R-1 (Cryptocapsin)	
	^13^C-NMR	^1^H-NMR	^13^C-NMR	^1^H-NMR
1	37.14		34.28	
2	48.42	1.48 (2H, m)	39.63	1.47 (2H, overlapped)
3	65.13	3.99 (1H, m)	19.26	1.64 (2H, m)
4	42.54	2.39 (1H, dd, 17.2, 4.8)	33.12	2.02 (2H, m)
5	126.25		129.50	
6	137.75		137.89	
7	125.53	6.12 (3H, s)	126.96	6.19 (1H, overlapped)
8	138.45	6.12 (3H, s)	137.71	6.14 (1H, overlapped)
9	135.91		136.53	
10	131.66	6.17(1H, d, 11.4)	131.71	6.71(1H, overlapped)
11	125.87	6.89 (1H, overlapped)	125.70	6.89 (1H, overlapped)
12	137.61	6.36 (1H, d, 15.2)	137.03	6.36 (1H, br. d, 14.5)
13	132.39		137.71	
14	132.00	6.26 (1H, d, 12.0)	132.17	6.26 (1H, br. d, 11.5)
15	131.24	6.15 (1H, overlapped)	130.75	6.15 (1H, overlapped)
16	28.74	1.07 (3H, s)	28.98	1.03 (3H, s)
17	30.27	1.26 (3H, s)	29.71	1.25 (3H, s)
18	21.63	1.74 (3H, s)	21.79	1.72 (3H, s)
19	12.79	1.99 (3H, s)	12.74	1.99 (3H, s)
20	12.96	1.96 (3H, s)	12.80	1.96 (3H, s)
1′	43.99		43.97	
2′	50.84	2.06 (1H, m)	50.84	2.00 (1H, m) 1.71 (1H, overlapped)
3′	70.38	4.52 (1H, m)	70.36	4.52(1H, m)
4′	45.29	1.51 (1H, overlapped) 2.96 (1H, dd, 14.4, 8.4)	45.29	1.49 (1H, overlapped) 2.96 (1H, dd, 14.0, 8.5)
5′	58.96		58.94	
6′	203.05		202.96	
7′	120.88	6.44 (1H, d, 15.0)	120.84	6.44 (1H, d, 15.0)
8′	146.95	7.33 (1H, d, 15.0)	146.89	7.33 (1H, d, 15.0)
9′	133.64		133.59	
10′	140.79		140.76	6.55 (1H, br, d, 10.0)
11′	124.10	6.62 (1H, overlapped)	124.04	6.62 (1H, overlapped)
12′	142.02		142.01	6.55 (1H, br. d, 10.0)
13′	136.13		135.80	
14′	135.28	6.36 (1H, d, 14.4)	135.31	6.36 (1H, br. d, 14.5)
15′	129.71	6.64 (1H, overlapped)	129.55	6.63 (1H, overlapped)
16′	25.08	1.21 (3H, s)	25.09	1.21 (3H, s)
17′	25.88	0.84 (3H, s)	25.86	0.84 (3H, s)
18′	21.32	1.36 (3H, s)	21.29	1.37 (3H, s)
19′	12.84	1.96 (3H, s)	12.85	1.98 (3H, s)
20′	12.93	1.96 (3H, s)	12.90	1.98 (3H, s)

^a^ Data (δ) were measured in CDCl_3_ at 500 MHz for ^1^H-NMR and 125 MHz for ^13^C-NMR. Coupling constants (*J*) in Hz are given in parentheses. Assignments were based on DEPT, ^1^H-^1^H COSY, HMBC and HSQC experiments.

**Table 2:** Comparison of main carotenoids content at different maturity stages of MT fruits.

	Capsorubin (Peak-1)	Capsanthin (Peak-2)	Lutein (Peak-3)	Cryptocapsin (Peak-4)	β-Crypto xanthin (Peak-6)	β-Carotene (Peak-7)
Unsaponified						
Immature	5.97 ± 0.22 ^b^	1.89 ± 0.08 ^a^	55.68 ± 0.85 ^b^	3.42 ± 0.15 ^a^	12.65 ± 0.17 ^a^	41.66 ± 1.48 ^b^
Premature	4.14 ± 0.03 ^a^	4.32 ± 0.23 ^b^	16.28 ± 0.57 ^a^	12.2 ± 0.61 ^b^	11.53 ± 0.07 ^a^	18.01 ± 0.61 ^a^
Fully mature	6.72 ± 0.02 ^d^	23.72 ± 0.55 ^c^	-	55.01 ± 1.51 ^d^	13.13 ± 1.18 ^b^	-
Overmature	6.33 ± 0.01 ^c^	30.55 ± 0.77 ^d^	-	15.52 ± 1.07 ^c^	13.12 ± 0.23 ^b^	-
Saponified						
Immature	3.34 ± 0.05 ^a^	1.37 ± 0.14 ^a^	26.25 ± 0.95 ^b^	4.07 ± 0.24 ^a^	7.97 ± 0.13 ^a^	15.1 ± 0.26 ^d^
Premature	3.27 ± 0.01 ^a^	14.69 ± 0.67 ^b^	18.85 ± 0.5 ^a^	34.68 ± 1.78 ^b^	10.23 ± 0.51 ^c^	9.9 ± 0.42 ^c^
Fully mature	6.42 ± 0.04 ^c^	57.65 ± 1.97 ^c^	-	171.66 ± 4.85 ^c^	9.38 ± 0.02 ^b^	4.55 ± 0.12 ^a^
Overmature	6.82 ± 0.04 ^d^	94.48 ± 1.59 ^d^	-	291.63 ± 13.4 ^d^	11.54 ± 0.22 ^d^	5.53 ± 0.1 ^b^

Not detected. Values (μg/g, dw) are mean ± standard deviation from three independent experiments. Peak no. see Figure 6. Different superscripts in the same column indicate significant differences (*p* < 0.05).

## Data Availability

The data that support the findings of this study are available from the corresponding author.

## Sample Availability

Samples of the compounds are available from the authors.
